# Compliance, Adherence and Concordance Differently Predict the Improvement of Uremic and Microbial Toxins in Chronic Kidney Disease on Low Protein Diet

**DOI:** 10.3390/nu14030487

**Published:** 2022-01-23

**Authors:** Andreana De Mauri, Deborah Carrera, Matteo Vidali, Marco Bagnati, Roberta Rolla, Sergio Riso, Massimo Torreggiani, Doriana Chiarinotti

**Affiliations:** 1 Nephrology and Dialysis Unit, Maggiore della Carità University Hospital, 28100 Novara, Italy; doriana.chiarinotti@maggioreosp.novara.it; 2 Nutritional Science and Dietetic, Maggiore della Carità University Hospital, 28100 Novara, Italy; deborah.carrera@libero.it (D.C.); sergio.riso@maggioreosp.novara.it (S.R.); 3 Clinical Chemistry Unit, Fondazione IRCCS Ca’ Granda Maggiore Policlinico Hospital, 20122 Milano, Italy; matteo.vidali@gmail.com; 4 Clinical Chemistry Laboratory, Maggiore della Carità University Hospital, 28100 Novara, Italy; marco.bagnati@maggioreosp.novara.it (M.B.); roberta.rolla@med.uniupo.it (R.R.); 5 Department of Health Sciences, Amedeo Avogadro University of Eastern Piedmont, 28100 Novara, Italy; 6 Néphrologie et Dialyse, Centre Hospitalier Le Mans, 72037 Le Mans, France; maxtorreggiani@hotmail.com

**Keywords:** low protein diet, chronic kidney disease, compliance, adherence, concordance, p-cresol, indoxyl-sulphate, lipoprotein-associated phospholipase A_2_

## Abstract

Background. In medicine, “compliance” indicates that the patient complies with the prescriber’s recommendations, “adherence” means that “the patient matches the recommendations” and “concordance” means “therapeutic alliance” between patient and clinician. While a low protein diet (LPD) is a cornerstone treatment of chronic kidney disease (CKD), monitoring the actual performance of LPD is a challenge. Patients. Fifty-seven advanced CKD adult patients were enrolled and LPD prescribed. Compliance was evaluated through the normalized protein catabolic rate (nPCR), adherence by the dietitian by means of a 24-h dietary recall and concordance by the nephrologist during consultations. Traditional parameters as well as total p-Cresyl Sulphate (t-PCS), total Indoxyl Sulphate (t-IS) and Lipoprotein-associated phspholipase A_2_ (Lp-PLA_2_) were compared between adherent/not adherent and concordant/not concordant subjects at enrolment and after two months. Results. nPCR, blood urea nitrogen, cholesterol and triglycerides significantly decreased in all patients. t-PCS and t-IS decreased among adherent subjects. Lp-PLA_2_, t-PCS, free-PCS and t-IS decreased among concordant subjects, while these increased in non-concordant ones. Conclusion. This study demonstrates that LPD may improve the control of traditional uremic toxins and atherogenic toxins in “adherent” and “concordant” patients. A comprehensive and multidisciplinary approach is needed to evaluate the compliance/adherence/concordance to LPD for optimizing nutritional interventions.

## 1. Introduction

Advances in pharmacologic therapy in the last five decades has allowed for the stabilization, functional recovery and favourable outcomes in patients with chronic diseases, provided that they have to assume their therapies their entire life. In this context, a new challenge for clinicians is how to verify whether patients follow medical prescriptions. This is a very old issue: even Hippocrates recorded whether patients took their medications to monitor the effects of the therapy. Yet, recently, the medical terminology has been evolving and incorporating new concepts [1].

The terms “compliance”, “adherence” and “concordance” are usually used as synonymous, but they indicate different ways of monitoring medical prescriptions.

As described by De las Cuevas, the word “compliance” firstly appeared in the English literature in 1640, meaning “the act of conforming, acquiescing, or yelding, … in a weak and subservient way”. In medicine, compliance indicates “the extent to which the patient’s behaviour matches the prescriber’s recommendations” [2]. Compliance has been criticized for its paternalistic and authoritarian meaning, implying that the subject passively “obeys” a clinician’s orders. In contrast, the word “adherence” appeared in 1530, with the meaning of “the quality of adherence, a steady devotion…”, and in medicine, adherence is defined as “the extent to which the patient’s behaviour matches agreed recommendations from the prescriber” [3], emphasizing just the free agreement of the patient to medical prescriptions from the doctor. Finally, the word “concordance” only appeared in 1997 and refers to the extent to which patients are successfully involved in shared decision making about their therapy [4,5]. Concordance means “therapeutic alliance”, “partnership”, a way to share information and agreed solutions between the patient and their healthcare provider.

Whereas compliance and adherence are relatively easy to measure, through several direct (observation, biological markers testing) or indirect (questionnaires, self-reports, diaries, anthropometric measures) tools, concordance is hard to quantify and cannot be empirically tested. It is often determined by the patient–doctor relationship in the light of their mutual knowledge, trust and attitude [6].

A cornerstone of long-life treatment of patients with advanced chronic kidney disease (CKD) is low protein diet (LPD), characterized by a protein intake of 0.6 to 0.2 g per kilogram of body weight per day, vegetable-enriched and sodium and phosphorus-depleted [7]. LPD is a safe and low-cost therapy, controls uremic symptoms and toxins generation, delays the progression to end stage kidney disease and seems to increase patients’ survival [8,9,10,11].

Uremic syndrome is a condition characterized both by a dysbiotic gut microbiota and accelerated atherosclerosis. The intestinal microbiota of CKD patients is characterized by the shift from saccharolytic species to proteolytic ones, which contribute to the generation of several toxins [12,13]. The most studied microbial-derived toxins are p-Cresyl Sulphate (PCS), derived from liver sulphatation of tyrosine and phenylalanine phenolic metabolites, and Indoxyl-Sulphate (IS), derived from liver sulphatation of tryptophan metabolite [12]. Both PCS and IS have been correlated with the progression of renal failure and cardiovascular morbidity and mortality in CKD [14,15,16]. The accelerated atherosclerosis is due to several mechanisms, among them Lipoprotein-associated phospholipase A_2_ (Lp-PLA_2_) plays a pivotal role. Lp-PLA_2_, a serine lipase produced by activated monocytes and macrophages, enters the vessel wall, catalyses the hydrolysis of LDLs phospholipids, induces the chemotaxis of leucocytes into the sub-intimal space and, eventually, contributes to the atherosclerotic plaque instability [17]. As matter of fact, Lp-PLA_2_ predicts acute cardiovascular events [18,19].

In this context, there are two important challenges in nephrology clinical practice: first, to determine whether an LPD could modulate the dysbiotic microbiota and atherogenic lipid profile and reduce uremic toxins in CKD patients and, second, how to verify whether patients follow renal nutritional prescriptions.

The aim of this study was, first, to assess and compare compliance, adherence and concordance among patients with advanced CKD on LPD and, second, to evaluate the impact of compliance, adherence and concordance on the reduction of the traditional, gut-derived and proatherogenic serum toxins.

## 2. Patients and Methods

### 2.1. Participants

Adult patients with advanced CKD not on dialysis (i.e., an estimated glomerular filtration rate (eGFR) between 6 and 25 mL/min/1.73 m^2^) afferent to the outpatient’s ambulatory division of Nephrology and Dialysis at Maggiore della Carità Hospital in Novara were eligible. Patients refusing to sign the informed consent or refusing LPD were excluded.

### 2.2. Intervention

Low protein diet composition: proteins 0.6 g/kg of “target” body weight/day, energy intake 25–30 kcal/kg/day, salt less than 6 g/day, phosphorus load less than 800 mg/day, low content of saturated fats and cholesterol, high content of fibres; calcium, vitamin D, folic acid, vitamin B12, iron, erythropoietin supplementation according to the usual clinical indications and good clinical practices [7]. Free-protein products were prescribed when needed.

Compliance to the diet was directly tested through 24 h urine collection in order to measure total urine nitrogen (TUN) excretion according to the Maroni–Mitch formula [20]:

TUN = urine urea (g/day) + 0.031 × body weight,

protein Catabolic Rate (PCR) according to the formula:

PCR = 6.25 × TUN (g/day)

and the total urine sodium excretion.

Adherence to the diet was verified by a trained dietitian by means of the dietary journal and through a dietary interview (24 h recall). The dietician calculated the protein-caloric intake and determined the discrepancy with the prescribed regimen. A difference greater than 30% was considered not adherence. The 30% threshold was arbitrarily chosen according to clinical experience and everyday practice.

Compliance to the diet was verified by a nephrologist through informal questions on prescribed drugs and nutritional therapy and knowledge about their contents and acknowledgement about mistakes or inobservance: a positive (concordant) or negative (no concordant) evaluation was assigned.

In order to motivate patients, during the first consultation, they were taught about the metabolic alterations secondary to advanced kidney disease and the effects of high protein intake on metabolic parameters and glomerular function. As a consequence, the potential benefits of an LPD were also discussed. Moreover, patients were instructed on how to weigh, cook and season food. In the following consultations, the nephrologist and the dietician posed some questions about the knowledge and the comprehension the patient had about the role of LPD in managing kidney disease. In particular, the examiner explored whether the patients understood why an LPD was proposed; the effect of LPD on bicarbonates, phosphates and CKD progression; and the willingness of the patient to pursue the low-protein regimen for their own benefit. Moreover, patients were asked about the effects of fibres in the diet and reassured about electrolyte imbalances (i.e., potassium intake). According to patient’s answers, arbitrarily, the trained evaluator was able to assign the patient to a group.

Biochemical parameters and nutritional assessment were compared between adherent/not adherent and concordant/not concordant patients. 

### 2.3. Study Design

At enrolment (T0) and after two months (T2), routinary laboratory measurements (haemoglobin, urea, creatinine, estimated glomerular filtration rate (eGFR) according to the CKD-EPI equation [21], sodium, potassium, uric acid, calcium, phosphate, parathyroid hormone (PTH), bicarbonates and albumin) were measured on an ADVIA^®^ 1800 Clinical Chemistry Analyzer (Siemens Healthcare Diagnostics, Munich, Germany); total and free serum p-Cresyl Sulphate (t- and f-PCS, respectively) and total and free serum Indoxyl Sulphate (t- and f-IS, respectively) were measured by means of a high-performance liquid chromatography technique coupled with tandem mass spectrometry (B.S.N. Srl, Castelleone, Italy); serum Lp-PLA_2_ activity was measured with the new PLAC^®^ test (Diazyme Laboratories, Inc., 12889 Gregg Court, Poway, CA, USA). 

Nutritional status was assessed by physical examination, measuring body weight, height, BMI (kg/m^2^), dominant Hand Grip strength (kg) using Hydraulic Hand Dynamometer Owner’s Manual (Sammons Preston, Bolingbrook, IL, USA), according to the reference values [22,23]. Bioelectrical impedance analysis (BIA) was used to estimate fat-free body mass (kg), fat mass (kg) and phase angle through an Akern model 101 (Akern Srl, Pisa, Italy).

### 2.4. Statistical Analysis

Statistical analyses were performed with the SPSS statistical software v.17.0 (SPSS Inc., Chicago, IL, USA) and R Language v.4.0.3 (R Foundation for Statistical Computing, Vienna, Austria). Normal distribution was preliminarily assessed by q-q plot and Shapiro–Wilk tests. Quantitative variables were expressed as mean and standard deviation while qualitative variables as absolute values and relative frequencies. Differences between groups for continuous variables were estimated by nonparametric Mann–Whitney U-test (for independent samples) and Wilcoxon Signed Ranks Test (for paired samples) or by parametric paired T-test. Concordance was verified by concordant pairs and Cohen’s kappa. A *p* < 0.05 was considered statistically significant.

## 3. Results

A total of 87 patients were assessed for eligibility: 27 were excluded (8 refused LPD, 7 refused to sign the informed consent, 12 did not satisfy the clinical criteria), 60 were enrolled and 57 subjects received the T2-evaluation (1 patient died and 2 patients were lost to follow-up). The mean age was 63.9 ± 11.8 years, 70% were males, 89% had arterial hypertension, 28% type II diabetes mellitus and 16% coronary artery disease

After 2 months of LPD, a significant reduction in BUN, total cholesterol and triglycerides was observed, without any difference with respect to the other biochemical and physical parameters. TUN and nPCR significantly decreased, according to the compliance to the diet (Table 1).

According to the dietitian’s interview, 41 (72%) patients resulted in being adherent to the diet, while, according to the nephrologist’s opinion, 34 (59%) patients were concordant with the nutritional therapy. Moreover, 30 (52%) and 11 (19%) patients received a positive and negative evaluation, respectively, from both the nephrologist and dietitian, with 73% being concordant pairs and a Cohen’s Kappa of 0.45, demonstrating a fair concordance.

### 3.1. Adherence Group

At T2, in comparison with T0, adherent patients showed reduced levels of nPCR, BUN, total cholesterol, triglycerides, LDL-Cholesterol, f-PC and f-IS and increased bicarbonates, while non-adherent subjects showed a statistical reduction in albumin levels (Table 2).

### 3.2. Concordance Group

At T2, in comparison with T0, concordant patients showed reduced levels of nPCR, BUN, total cholesterol, LDL-Cholesterol, Lp-PLA2, t-PC, f-PC and t-IS and increased bicarbonates. In contrast, non-concordant subjects showed increased levels of Lp-PLA2 and t-PC (Table 3).

### 3.3. Changes in Nutritional Status

Enrolled patients had, on average, a good nutritional status and no significant variations were observed in both adherent and concordant groups (Table 2 and Table 3).

## 4. Discussion

This study demonstrates that LPD reduces blood urea nitrogen, cholesterol and triglycerides and increases bicarbonates in all compliant patients that LPD reduces t-PC and T-IS in “adherent” ones and, finally, that LPD reduces gut-derived toxins (t-PC, f-PC, t-IS) and Llp-PLA_2_ in “concordant” patients, whereas an increased burden of uremic toxins was observed among non-concordant patients. This is in accordance with the literature, where every reduction of 0.2 g/kg of daily protein intake improves the progression of renal disease, independently on the initial values [24].

Protein-restricted diets are known to have an important role in reducing “traditional” uremic toxins responsible for uremic symptoms, such as ammonium solutes, controlling calcium-phosphorus metabolism alterations, sodium and water retention and electrolytes imbalances. An LPD also counteracts the endogenous protein catabolism and malnutrition, delays the progression to end-stage kidney disease and dialysis initiation and seems to improve the survival of CKD patients [8,9,10,11,25,26].

At the intestinal level, LPD is able to modulate the dysbiotic microbiota through several mechanisms: first, the reduction in animal proteins and amino acid intake limits the availability of substrates for proteolytic bacteria; second, the high content of vegetables, legumes, fibers and whole grains that are rich in prebiotics promotes the restoration of the saccharolytic pathway over the proteolytic one [27,28]. As largely discussed by Camerotto and colleagues, an LPD is characterized by a high content of fibers, on average 7.6 g/1000 Kcal for a traditional LPD, up to 11.6 g/1000 Kcal for a very-LPD or 16 g/1000 Kcal for a vegan LPD [29]. Protein-free products contain much more fibers than regular products: 4.8 vs. 2.7 g/100 g in protein-free and regular pasta, respectively, and 10.8 vs. 2.7 g/100 g in protein-free and regular bread, respectively [29]. Moreover, in more recent years, protein-free products are even more enriched with fibers, fructooligosaccharides, galactooligosaccharides, inulin and mannans, with an increased mean content from 4.2 to 10.8 g/100 g and from 1.5 to 4.8 g/100 g in bread and pasta, respectively [29]. As a matter of fact, Black et al. observed a reduction in PCS in CKD subjects after 6 months of LPD, while Marzocco et al. found a reduction in IS levels among CKD subjects after only 1 week of very low protein diet [30,31]. According to these findings, we observed a reduction in PCS and IS in adherent and concordant subjects.

In the present trial, an LPD also reduced Lp-PLA_2_ serum levels. Previous studies demonstrated that plant-based diets reduce the atherogenic lipid levels in the general population and, in particular, Lp-PLA_2_ [32,33,34]. However, although we could not ascertain the direct relationship between an LPD and Lp-PLA_2_, our study is the first to show a possible benefit of an LPD in reducing Lp-PLA_2_ levels in patients with advanced renal failure. Lp-PLA_2_ is an emergent proatherogenic molecule, predicting cardiovascular disease and mortality in the general population as well as in cardiac or diabetic patients, so that the three major heart international societies, the American Heart Association, the American College of Cardiology and the European Society of Cardiology, include the Lp-PLA_2_ activity determination in the risk stratification charts in order to optimize the lipid lowering treatment [35]. Some evidences show that, in uremic subjects, serum Lp-PLA_2_ values are elevated and predict acute cardiovascular events [36,37].

The main findings of this study are the differences observed in compliant, adherent and concordant patients: the deeper and more comprehensive the investigation is, the earlier and more accurate are the results. Adherence to the medical prescriptions is recognized to improve healthcare-related outcomes, and in the nephrology setting, poor dietary “adherence” is associated with worse outcomes [38,39]. Several studies investigated different tools for testing adherence to renal diets, such as objective (blood and urinary parameters) and subjective approaches (24-h or 3 day food recalls, food frequency questionnaires) or a combination of the two. Indeed, in our study, we used blood and urinary parameters to test “compliance” and the 24 h recalls were assessed by the dietitian to test “adherence”. 

As a consequence of the different definitions, the literature provides highly variable rates of adherence, ranging from about 9 to 50% [40]. However, we found even greater figures: 72% of subjects are recognized as “adherent”, 59% as “concordant” and 52% received a positive judgment from both the dietitian and the nephrologists.

Among several factors associated with adherence, such as age, educational level and family support, the role of the “knowledge” is controversial with either positive or negative association, but the literature, in general, agrees that the patient “activation” and “engagement” are needed to ameliorate adherence [40,41,42,43]. Based on these considerations, in the present study, we tried to convert the subjective and unmeasurable concept of “concordance” into a semi-quantitative variable (presence/absence) in order to obtain objective results (variations in blood and urinary parameters). Concordance was judged by the nephrologist based on the mutual acquaintance with the patient because the quality of the relationship between the healthcare provider and the patient is recognized as an important modifier of therapy adherence [40]. Nevertheless, as well as for the nutritional and pharmacological prescription and for the feedback, the combined approach of nephrologists and dietitians is advisable because the multidisciplinary team care has been demonstrated to protect from the progression of renal disease [44].

The main limitation of this study is the low sample size and the short follow-up: Several larger controlled trials are needed to observe an eventual benefit on renal and patients’ survival. Moreover, in addition to the terms “adherence” and “concordance”, the term “persistence”, describing the duration of continuous medication use, is becoming more and more important for patients with chronic diseases [45]. In this respect, nutritional therapy being a life-long therapy for CKD patients, regardless of the stage of kidney disease, dialysis or kidney-transplantation status, further studies are needed to assess persistence in the nutritional approach system of care.

In conclusion, the present study demonstrates that a comprehensive approach to evaluate compliance, adherence and concordance to an LPD in CKD patients is useful to monitor and predict nutritional interventions and timely adapt them to guarantee the persistence of the patients in the system of care. In the modern era of “personalized” medicine, and particularly in the context of more “personalized” renal diets, a “personalized” partnership between the doctor and the patient is also desirable in order to optimize the nutritional therapy outcomes.

## Figures and Tables

**Table 1:** Comparison between biochemical and nutritional parameters and pharmacological therapy in all patients.

	T0	T2	*p*
EPI-CKD (mL/min)	18.1 ± 3.7	18.2 ± 4.7	0.77
Daily urine proteins (g/24 h)	1.58 ± 1.38	1.74 ± 1.96	0.31
Hemoglobin (g/dL)	12.0 ± 1.5	11.9 ± 1.5	0.08
**BUN (mg/dL)**	**52 ± 17**	**46 ± 15**	**0.007**
Uric acid (mg/dL)	6 ± 1.4	6 ± 1.2	0.49
Albumin (mg/dL)	4.2 ± 0.3	4.1 ± 0.3	0.07
Calcium (mg/dL)	9.1 ± 0.5	9.1 ± 0.5	0.19
Phosphorus (mg/dL)	3.7 ± 0.7	3.7 ± 0.8	0.59
**Total cholesterol (mg/dL)**	**186 ± 42**	**161 ± 70**	**0.004**
HDL (mg/dL)	45 ± 13	45 ± 13	0.50
**Triglycerides (mg/dL)**	**196 ± 151**	**161 ± 70**	**0.037**
LDL (mg/dL)	105 ± 37	94 ± 30	0.09
**HCO_3_ (mEq/L)**	**22.6 ± 3.2**	**23.6 ± 2.6**	**0.001**
PTH (ng/mL)	92.9 ± 76.4	97.5 ± 57.9	0.08
Urine Natrium (mEq/day)	144 ± 59	145 ± 60	0.47
Epoetin rensponse index (IU/gHb)	134 ± 345	124 ± 324	0.79
Epoetin zeta (IU/week)	1368 ± 3410	1280 ± 3143	0.81
Furosemide (mg/day)	38.2 ± 69.9	30.7 ± 45.5	0.41
**TUN g/kg/24 h**	**10.9 ± 3.5**	**9.5 ± 2.7**	**0.0001**
**nPCR (g/kg/day)**	**0.91 ± 0.3**	**0.77 ± 0.2**	**0.005**
Lp-PLA_2_ (nmol/mL/min)	165.5 ± 44.4	161.1 ± 45.8	0.52
t-PC (mcMol)	135.3 ± 78.4	120.4 ± 69.8	0.35
f-PC (mcMol)	5.21 ± 3.89	4.2 ± 3.1	0.08
t-IS (mcMol)	30.5 ± 14.6	28.4 ± 14.4	0.16
f-IS (mcMol)	1.44 ± 0.82	1.35 ± 0.99	0.52
BMI (kg/cm^2^)	29.4 ± 8.3	29.3 ± 8.5	0.38
Free Fat Mass (kg)	52.7 ± 11.8	52.7 ± 11.5	0.99
Fat Mass (kg)	23.7 ± 8.4	23.3 ± 8.7	0.31
Angle Phase	4.89 ± 1.11	4.80 ± 1.90	0.23
Hand Grip (kg)	33.8 ± 10.4	34.6 ± 10.3	0.15

In bold, significant values.

**Table 2:** Comparison between biochemical and nutritional parameters and pharmacological therapy in non-adherent and adherent patients.

	Non-Adherent			Adherent		
	T0	T2	*p*	T0	T2	*p*
EPI-CKD (mL/min)	16.6 ± 3.2	16.5 ± 4.6	0.86	18.6 ± 3.8	19.0 ± 4.6	0.53
Daily urine proteins (g/24 h)	1.9 ± 1.3	2.0 ± 2.1	0.66	1.5 ± 1.4	1.9 ± 1.9	0.26
Hemoglobin (g/dL)	11.4 ± 1.0	11.2 ± 1.2	0.15	12.2 ± 1.7	12.1 ± 1.5	0.18
**BUN (mg/dL)**	58 ± 23	52 ± 18	0.21	**50 ± 13**	**44 ± 14**	**0.017**
Uric acid (mg/dL)	6.1 ± 1.2	6.0 ± 1.1	0.46	6.0 ± 1.5	6.0 ± 1.3	0.75
**Albumin (mg/dL)**	**4.2 ± 0.3**	**4.0 ± 0.3**	**0.04**	4.3 ± 0.4	4.2 ± 0.4	0.37
Calcium (mg/dL)	9.0 ± 0.6	8.9 ± 0.5	0.61	9.1 ± 0.5	9.1 ± 0.6	0.26
Phosphorus (mg/dL)	3.7 ± 0.7	3.8 ± 0.8	0.75	3.6 ± 0.7	3.7 ± 0.8	0.79
**Total cholesterol (mg/dL)**	183 ± 61	171 ± 44	0.61	**186 ± 34**	**171 ± 30**	**0.002**
HDL (mg/dL)	47 ± 13	46 ± 14	0.28	45 ± 14	45 ± 13	0.99
**Triglycerides (mg/dL)**	161 ± 61	151 ± 42	0.61	**209 ± 172**	**165 ± 79**	**0.05**
**LDL (mg/dL)**	106 ± 45	94 ± 34	0.97	**105 ± 34**	**94 ± 29**	**0.037**
**HCO_3_ (mEq/L)**	23.0 ± 2.7	22.5 ± 2.6	0.63	**22.4 ± 3.4**	**24.0 ± 2.5**	**0.0001**
PTH (ng/mL)	113.8 ± 105.6	104.8 ± 58.9	0.49	**82.3 ± 63.4**	**94.2 ± 59.0**	**0.05**
Urine Natrium (mEq/day)	150 ± 60	148 ± 47	0.28	140 ± 58	144 ± 65	0.96
Epoetin response index (IU/gHb)	71 ± 196	119 ± 234	0.49	160 ± 368	132 ± 381	0.41
Epoetin zeta (IU/week)	800 ± 2242	1200 ± 2306	0.59	1609 ± 3718	1341 ± 3449	0.55
Furosemide (mg/day)	50.0 ± 81.3	43.3 ± 64.4	0.25	34.7 ± 66.7	26.8 ± 36.7	0.78
**nPCR (g/kg/day)**	0.82 ± 0.33	0.75 ± 0.22	0.15	**0.93 ± 0.28**	**0.78 ± 0.17**	**0.003**
Lp-PLA2 (nmol/mL/min)	158.2 ± 43.4	161.0 ± 54.1	0.97	167.5 ± 45.5	159.98 ± 42.9	0.15
t-PC (mcMol)	173.6 ± 90.2	151.5 ± 78.5	0.92	123.8 ± 71.1	109.7 ± 65.2	0.15
**f-PC (mcMol)**	7.49 ± 5.63	5.84 ± 4.52	0.86	**4.51 ± 2.81**	**3.63 ± 2.31**	**0.04**
t-IS (mcMol)	30.6 ± 10.1	30.2 ± 15.7	0.97	30.1 ± 16.0	27.9 ± 14.3	0.06
**f-IS (mcMol)**	1.56 ± 0.79	1.51 ± 1.10	0.91	**1.41 ± 0.83**	**1.31 ± 0.97**	**0.05**
BMI (kg/cm^2^)	30.5 ± 14.1	30.6 ± 14.4	0.73	29.0 ± 5.0	28.9 ± 5.0	0.19
Free Fat Mass (kg)	49.1 ± 15.7	49.4 ± 16.0	0.72	54.1 ± 10.0	53.9 ± 9.3	0.81
Fat Mass (kg)	21.2 ± 8.8	20.4 ± 9.0	0.35	24.5 ± 8.3	24.3 ± 8.5	0.57
Angle Phase	4.32 ± 1.44	4.29 ± 1.45	0.81	5.06 ± 0.92	4.98 ± 0.88	0.23
Hand Grip (kg)	31.0 ± 8.5	33.1 ± 9.6	0.1	35.0 ± 10.8	35.1 ± 10.6	0.66

In bold, significant values.

**Table 3:** Comparison between biochemical and nutritional parameters and pharmacological therapy in non-concordant and concordant patients.

	Non-Concordant			Concordant		
	T0	T2	*p*	T0	T2	*p*
EPI-CKD (mL/min)	18.1 ± 2.9	17.7 ± 4.2	0.64	18.8 ± 4.2	18.6 ± 5.1	0.52
Daily urine proteins (g/24 h)	1.9 ± 1.6	2.0 ± 2.3	0.91	1.49 ± 1.2	2.7 ± 2.4	0.68
Hemoglobin (g/dL)	11.7 ± 1.4	11.5 ± 1.6	0.34	12.2 ± 1.6	12.1 ± 1.4	0.12
**BUN (mg/dL)**	52 ± 20	50 ± 15	0.85	**52 ± 14**	**44 ± 14**	**0.003**
Uric acid (mg/dL)	6.4 ± 1.1	6.2 ± 1.1	0.28	5.8 ± 1.5	5.8 ± 1.3	0.99
Albumin (mg/dL)	4.1 ± 0.3	4.1 ± 0.3	0.48	4.3 ± 0.4	4.2 ± 0.4	0.1
Calcium (mg/dL)	8.9 ± 0.6	8.9 ± 0.5	0.96	9.2 ± 0.6	9.1 ± 0.6	0.13
Phosphorus (mg/dL)	3.7 ± 0.7	3.8 ± 0.8	0.41	3.6 ± 0.7	3.6 ± 0.9	0.98
Total cholesterol (mg/dL)	190 ± 53	177 ± 41	0.37	**183 ± 32**	**167 ± 29**	**0.005**
HDL (mg/dL)	46 ± 15	46 ± 14	0.6	45 ± 12	45 ± 12	0.8
Triglycerides (mg/dL)	198 ± 152	160 ± 53	0.11	193 ± 152	162 ± 80	0.14
LDL (mg/dL)	106 ± 47	99 ± 32	0.97	**104 ± 30**	**91 ± 28**	**0.03**
HCO_3_ (mEq/L)	22.6 ± 2.9	23.2 ± 2.9	0.18	**22.6 ± 3.4**	**23.9 ± 2.5**	**0.006**
PTH (ng/mL)	107.8 ± 87.8	104.8 ± 58.9	0.18	82.9 ± 67.1	93 ± 57.6	0.19
Urine Natrium (mEq/day)	164 ± 69	165 ± 56	0.92	133 ± 49	132 ± 55	0.37
Epoetin response index (IU/gHb)	150 ± 277	189 ± 455	0.78	160 ± 388	132 ± 361	0.49
Epoetin zeta (IU/week)	1608 ± 2965	1869 ± 4267	0.71	1205 ± 3715	882 ± 2056	0.46
Furosemide (mg/day)	38.0 ± 69.8	39.1 ± 57.9	0.79	38.2 ± 71.3	25.0 ± 34.7	0.19
**nPCR (g/kg/day)**	0.87 ± 0.27	0.81 ± 0.21	0.22	**0.93 ± 0.32**	**0.74 ± 0.19**	**0.002**
**Lp-PLA2 (nmol/mL/min)**	**175.6 ± 48.1**	**182.3 ± 48.6**	**0.019**	**158.9 ± 41.4**	**146.8 ± 38.2**	**0.013**
**t-PC (mcMol)**	**133.1 ± 79.1**	**141.1 ± 66.7**	**0.005**	**136.7 ± 79.1**	**106.9 ± 69.5**	**0.001**
**f-PC (mcMol)**	5.32 ± 4.46	4.84 ± 3.57	0.29	**5.13 ± 3.53**	**3.77 ± 2.91**	**0.003**
**t-IS (mcMol)**	29.4 ± 13.4	29.4 ± 13.3	0.32	**31.2 ± 15.5**	**27.7 ± 15.5**	**0.021**
f-IS (mcMol)	1.39 ± 0.64	1.23 ± 0.57	0.70	1.46 ± 0.92	1.43 ± 1.19	0.09
BMI (kg/cm^2^)	28.3 ± 3.9	28.4 ± 3.9	0.91	30.1 ± 10.2	29.9 ± 10.4	0.29
Free Fat Mass (kg)	54.4 ± 10.4	54.9 ± 10.2	0.31	51.7 ± 12.7	51.3 ± 12.2	0.28
Fat Mass (kg)	24.5 ± 7.6	24.2 ± 6.7	0.54	23.7 ± 8.1	2.4 ± 9.2	0.41
Angle Phase	4.68 ± 0.85	4.71 ± 0.93	0.85	5.14 ± 0.90	5.00 ± 0.85	0.11
Hand Grip (kg)	31.5 ± 3.6	31.8 ± 4.2	0.21	32.1 ± 4.2	31.5 ± 4.5	0.49

In bold, significant values.

## Data Availability

The data presented in this study are available on request from the corresponding author.
